# Heparin-Induced Thrombocytopenia in Healthy Individuals with Continuous Heparin Infusion

**DOI:** 10.1055/s-0038-1624565

**Published:** 2018-01-30

**Authors:** Jonathan S. Williams, Paula J. Autori, Stephen K. Kidd, Gregory Piazza, Molly C. Connors, Charles A. Czeisler, Karine D. Scheuermaier, Jeanne Duffy, Elizabeth B. Klerman, Frank A. Scheer, Marjorie Kozak, Sheila M. Driscoll, Samuel Z. Goldhaber

**Affiliations:** 1 Division of Endocrinology, Diabetes, and Hypertension, Brigham and Women's Hospital, Boston, Massachusetts, United States; 2 Center for Clinical Investigations, Brigham and Women's Hospital, Boston, Massachusetts, United States; 3 Department of Cardiology, Northwestern Memorial Hospital, Chicago, Illinois, United States; 4 Cardiovascular Division, Brigham and Women's Hospital, Boston, Massachusetts, United States; 5 Division of Sleep Medicine, Brigham and Women's Hospital, Boston, Massachusetts, United States; 6 Wits Sleep Laboratory, University of the Witwatersrand, Johannesburg, Braamfontein, South Africa

**Keywords:** healthy volunteer, heparin, platelets, thrombocytopenia

## Abstract

The risk for developing heparin-induced thrombocytopenia in healthy individuals is thought to be low, but monitoring recommendations remain controversial. Therefore, a retrospective cohort study was conducted to identify the incidence of thrombocytopenic events in a healthy research population exposed and re-exposed to continuous intravenous (IV) unfractionated heparin. The Division of Sleep Medicine and the Centre for Clinical Investigations at Brigham and Women's Hospital, Boston, Massachusetts, United States, instituted a standardized platelet monitoring procedure for all research protocols that involved heparin to detect platelet count decreases. Protocol-related frequent blood sampling required use of continuous IV unfractionated heparin infusion (5,000 unit/L in 0.45% saline at 40 mL/h) to maintain line patency over extended periods of IV access. From the years 2009 to 2012, a total of 273 healthy volunteers enrolled in Sleep Medicine research protocols met study criteria as having been exposed and/or re-exposed to continuously infused intravenous heparin for at least 4 hours. The mean continuous heparin exposure time was 88 ± 82 SD hours with a total of 397 heparin exposure and re-exposure events. Platelet count measurements were obtained on 629 occasions, representing a range from 2 to 9 draws per participant. No platelet count decrease of more than 50% was detected. There were no detected adverse bleeding or thrombotic events. In this retrospective study of healthy volunteers involved in a rigorously applied inpatient platelet monitoring protocol, heparin exposure and re-exposure did not lower platelet concentration and, therefore, does not appear to be associated with increased risk of HIT in this population.

## Introduction

Heparin-induced thrombocytopenia (HIT) is a prothrombotic immune reaction to unfractionated and low-molecular-weight heparin (LMWH) products mediated by platelet-activating antibodies against complexes of platelet factor 4 (PF4) and heparin. 1 2 Though venous thromboembolism is most common in medical patients, arterial thromboembolism can occur and is associated with an increased risk of mortality and morbidity, including limb ischemia, amputation, and stroke. 1 HIT is suspected when the baseline platelet count decreases by 50%. Case detection of HIT is based on three recognized types of presentation: (1) typical-onset thrombocytopenia (∼65% of HIT cases) that occurs between 4 and 14 days of exposure, (2) rapid-onset thrombocytopenia (∼30%) that occurs within 100 days of heparin re-exposure and leads to abrupt platelet decrease in less than 24 hours, and (3) delayed-onset thrombocytopenia (∼5%) that occurs within a median of 9 days (range: 9–40 days) following discontinuation of heparin.

It is postulated that the development of HIT requires additional patient-specific factors related to comorbid illness or severity of disease. 3 4 The incidence is up to 10 times higher among patients receiving unfractionated heparin versus LMWH. 3 5 HIT occurs more frequently among patients who have had major surgery (especially cardiac surgery) than among patients who have had minor surgery 3 5 or those only receiving medical therapy. 6 It is rare in otherwise healthy obstetrical patients, although outside of pregnancy, women are at slightly higher risk than men. 6

Recommendations regarding platelet monitoring for case detection take into consideration that excessive testing for HIT antibodies is common. 7 8 9 Efforts to reduce unnecessary testing include the American Society of Haematology *Choosing Wisely* campaign. 10 The 4T score is a validated pre-test probability tool to screen for HIT in hospitalized patients. As the negative predictive value for 4T is very high, additional testing for HIT in patients with a low score can safely be avoided. The American College of Chest Physicians (ACCP) recommends routine monitoring of platelet counts to detect the first two types of presentations described earlier, but not following heparin cessation. 11

The risk of developing clinical HIT in healthy individuals is thought to be low, but monitoring recommendations remain controversial. 12 Therefore, we investigated serial platelet count measurements in healthy volunteers participating in research trials that required prolonged and repeated exposure to continuously infused intravenous unfractionated heparin to determine if significant platelet reductions were detected.

## Methods

We conducted a retrospective cohort study of subjects participating in Brigham and Women's Hospital/Harvard Medical School Division of Sleep Medicine inpatient research protocols from January 2009 through December 2012. All research protocols included continuously infused intravenous heparin.

## Heparin Infusion and Platelet Count Monitoring Protocol

**Fig. 1:**
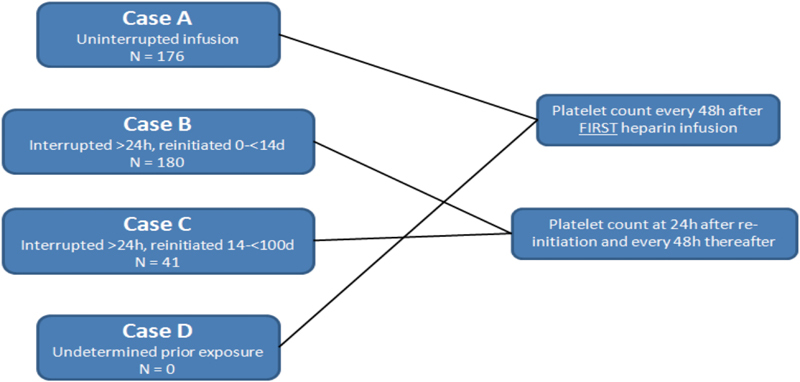
Platelet monitoring algorithm based on prior heparin exposure. Case A represents a continuous heparin infusion, with platelet counts monitored every 48 hours. Case B represents a stop in heparin infusion > 24 hours and <14 days. Case C represents a stop in heparin infusion followed by a restart after 14 days. Case D was unknown exposure history.

Continuous unfractionated heparin infusion (5,000 unit/L in 0.45% saline at 40 mL/h [i.e., 200 unit/h]; Hospira Inc, Lake Forest, Illinois, United States) was introduced to prevent clot formation during blood sampling through long (12 ft) peripheral intravenous (IV) lines, which were required to permit frequent blood sampling without disruption of sleep patterns. Unfractionated heparin was prepared by the Brigham and Women's Hospital Investigational Drug Service. Due to concern for inducing HIT, a standard operating procedure (SOP) was created in 2007 for platelet count monitoring in all protocols (see Supplementary Material ). Monitoring algorithms were based on heparin exposure and re-exposure time points. Platelet count measurements were obtained every 48 hours, and the duration of testing was adjusted for concern of immediate versus delayed thrombocytopenic risk ( Fig. 1 ).

## Study Population

All participants were screened for eligibility in individual research protocols with medical history, physical examination, laboratory testing, and detailed psychological review. 13 14 All study protocols required potential participants to have (1) no personal history of chronic illnesses (i.e., hypertension, diabetes, asthma, or any vascular disease); (2) no hospitalizations in the past year; (3) no major surgical history or surgery that resulted in permanent artificial hardware (joint replacement, plates, screws, or pins); (4) no use of prescription medications; (5) no recent use of tobacco, opiates, or illegal drugs; and (6) no recent alcohol use. A normal detailed physical screening examination was required, and normal laboratory values were required for (1) complete blood count, (2) comprehensive metabolic panel, (3) urinalysis, (4) thyroid function tests, (5) toxicology screening, and (6) electrocardiogram.

## Study Protocol

Research protocol inpatient study visit duration during the study years varied from 2 to 72 days. Common elements included single-occupancy rooms, standardized SOPs for procedures, and multiday IV catheters for frequent blood sampling during both wake and sleep periods. The platelet count monitoring protocol was developed based on the American College of Chest Physicians (ACCP) guidelines. 15 The Partners Healthcare System (Brigham and Women's Hospital/Massachusetts General Hospital) Institutional Review Board approved the described project and all protocols included. Written informed consent, including the risk of HIT with heparin infusion, was obtained from all study participants for each protocol.

## Analytical Plan

A heparin exposure event was counted when an infusion continued, uninterrupted, for at least four hours. A heparin “re-exposure” event was defined as stopping an infusion after at least 4 hours of continuous infusion and restarting the infusion at a later date. The total heparin exposure in hours was tallied for each participant, along with the number of heparin start-stop events (re-exposures). Blood draws for platelet count monitoring occurred as per the SOP ( Fig. 1 ). A paired sample one-sided *t* -test was used to compare within subject changes in platelet values at each draw point from baseline. A clinically significant decrease in platelet count was defined as a decrease from baseline of more than 50% which would trigger an investigation for HIT as per the SOP. Platelet count measurements at less than 96 hours in volunteers without prior heparin exposure were not included in the analysis of platelet count distribution. Neither PF4 antibody ELISA nor serotonin release assay testing was undertaken.

## Results

**Fig. 2:**
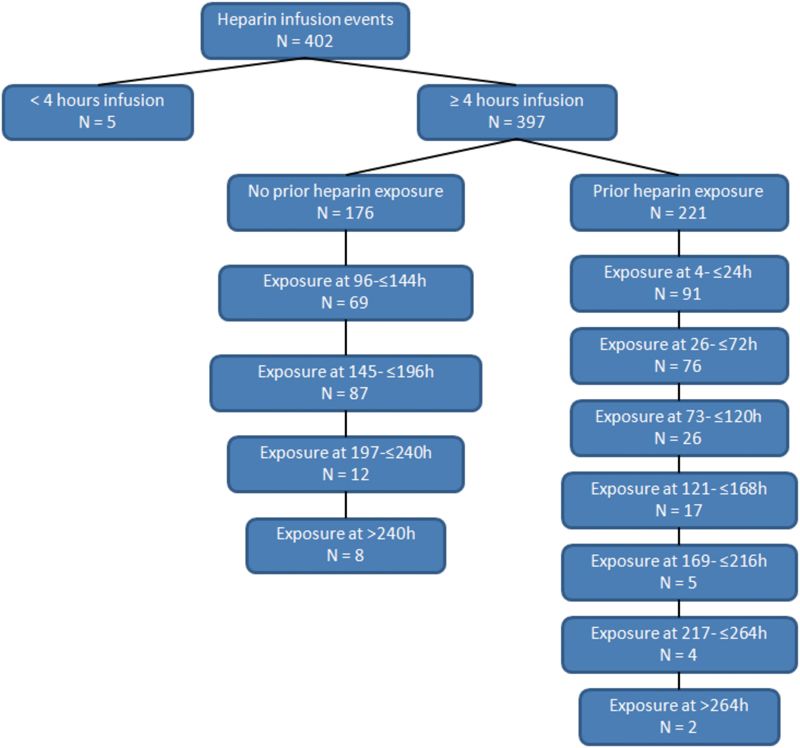
Number of heparin infusion events, including duration and number of re-exposure events.

A total of 273 participants providing 402 individual heparin exposure events (initial and re-exposures) were identified. Of these heparin exposure events, 397 exposures of more than 4 hours qualified for platelet monitoring ( Fig. 2 ). Most participants were young and Caucasian with a slight male predominance ( Table 1 ). All volunteers had normal hemoglobin and platelet count measurements at baseline.

**Table 1:** Baseline participant demographics of normal healthy volunteers

Category	*N* (%)
Total subjects	273
Age (y)	29.8 ± 12.0
Gender	
Male	145 (53)
Female	128 (47)
Race	
Caucasian	202 (74)
African American	30 (11)
Asian	29 (11)
Hispanic	12 (4)
Weight (kg)	70.5 ± 11.3
Screening hemoglobin (g/dL)	Female: 12.5 ± 1.0 Male: 14.6 ± 1.0
Baseline platelet count (×1,000/mL)	237 ± 55

Note: Mean ± standard deviation.

The baseline platelet count in all participants averaged 237 ± 55 (×1,000/mL). A total of 629 platelet count measurements were recorded at times varying from 24 to greater than 264 hours after initiation of the heparin infusion. At 96 hours, the mean platelet count was 234 ± 49 (×1,000/mL; *p* = 0.07) and at 240 hours, 254 ± 13 (×1,000/mL; *p* =  > 0.9). The distribution of platelet count values based on time from heparin exposure is depicted in Table 2 . Longer protocols required longer total heparin exposure time, more frequent re-exposure events, and more blood draws for platelet monitoring ( Fig. 2 ). The mean duration of heparin infusion was 88 ± 82 hours with a maximum of 666 hours.

**Table 2:** Platelet count values by time from initial heparin exposure

	Platelet sampling						
Hours since initiation of infusion	24 h	72 h	96 h	144 h	192 h	240 h	≥288 h
Platelet count (k/mL)	246 ± 56 ( *p* = > 0.9)	232 ± 47 ( *p* = 0.12)	234 ± 49 ( *p* = 0.07)	237 ± 48 ( *p* = 0.88)	243 ± 5 ( *p* = > 0.9)	254 ± 13 ( *p* = > 0.9)	241 ± 43 ( *p* = > 0.9)
Total blood draws *N* = 629	91	76	265	141	30	13	13

Note: Mean ± standard deviation.

There were no cases where serial platelet counts decreased more than 50% from baseline (discussed previously). Furthermore, no arterial or venous thromboembolic events occurred in this cohort of healthy research volunteers.

## Discussion

We found no indication of HIT with serial platelet count monitoring. Historically, bovine lung heparin often increased the risk of HIT, even in healthy individuals. 16 In the contemporary era using porcine gut heparin, healthy, non-smoking individuals without prior heparin exposure rarely develop PF4 antibodies when exposed to UFH or LMWH. 4 The frequency of HIT in medical service patients receiving unfractionated heparin is estimated at 0.1 to 1%. 12

Whether patients with an estimated HIT risk of less than 1% should undergo routine platelet count monitoring while on unfractionated heparin infusion remains controversial. 12 This study suggests that routine serial platelet count monitoring in healthy research volunteers who are undergoing continuous heparin infusion may be unnecessary. This same low risk of HIT may apply to a select group of medical service patients with low disease severity and few comorbid diseases, though further investigation of this population is needed.

The classic diagnostic algorithm to assess the pre-test probability of HIT and guide subsequent PF4 antibody ELISA and serotonin release assay (SRA) testing involves scoring the “4Ts”: (1) degree of *t* hrombocytopenia, (2) *t* iming with respect to heparin exposure, (3) evidence of *t* hrombosis, and (4) likelihood of alternative causes of *t* hrombocytopenia. 2 Women and surgical patients, particularly those with severe trauma, are at higher risk of HIT. Additional medical comorbidities likely increase risk, though the magnitude of risk remains uncertain. 3 4 17 Our findings suggest that healthy volunteers appear to have a low risk of HIT.

This study is limited by the relatively small sample size, which limits the power to detect HIT in this low incidence (<1%) population. A second limitation is that no additional assays were utilized to confirm or exclude HIT. Strengths of this study include that uniform procedures were utilized to assess participant eligibility, heparin concentration, and platelet count monitoring at fixed predefined intervals that addressed both typical- and delayed-onset forms of HIT presentation.

## Conclusion

In summary, heparin exposure and re-exposure in our study population of healthy individuals did not lower absolute platelet counts, the usual initial laboratory abnormality in HIT. Our study suggests that routine platelet count monitoring in healthy research subjects or medical service patients with a low risk of developing HIT and who are undergoing continuous heparin administration may not be necessary.
