# Supplementary material for: Heparin-Induced Thrombocytopenia in Healthy Individuals with Continuous Heparin Infusion
Source: TH Open. 2018 Jan 30;2(1):e49–53. doi: 10.1055/s-0038-1624565 (PMC6524859; doi:10.1055/s-0038-1624565)
Supplement: Supplementary file 1 — Supplementary Material [file 10-1055-s-0038-1624565-s170012-1.pdf]

Blood Sample Collection for Platelet Counts for Heparin-Induced Thrombocytopenia Surveillance

Purpose

To establish a standard operating procedure for collection and measurement of platelet counts to screen for heparin-induced thrombocytopenia

Equipment

Platelet Screening

- Standard blood drawing apparatus.
- 3-mL syringe.
- 3-mL EDTA tube.
- Brigham and Women's Hospital Clinical Laboratory Test Requisition Form.
- Biohazard Specimen Bag.

Platelet Factor-4 Antibody Test

- Standard phlebotomy equipment.
- 3-mL syringe.
- 3-mL citrate tube.
- Brigham and Women's Hospital Clinical Laboratory Test Requisition Form.
- Biohazard Specimen Bag.

Responsibility

It is the responsibility of the physician conducting the pre-study physical examination to document the most recent use of heparin in a subject. If heparin has been initiated in the 100-day window before the subject's admission to the research center, it is the responsibility of the screening physician to document the date at which heparin was FIRST initiated in that subject (day 0).

It is the responsibility of the research project leader to DOCUMENT on the orders at the beginning of the heparin order: (1) the baseline platelet level obtained from screening, (2) whether heparin has been INITIATED in the 100-day window before the subject's admission, and if so (3) to document the date of first initiation of heparin (day 0) in that 100-day window.

It is the responsibility of the attending physician to VERIFY that all the above information has been provided by the research project leader (1—baseline platelet level, 2—whether heparin has been initiated in the 100-day window before subjects' admit, and 3—documentation of the date of first heparin initiation, day 0) and with this information, to determine which case is to be applied when the subject receives heparin for the first time in the study.

It is the responsibility of certified blood-drawing personnel to collect, aliquot, transport, and document blood samples for platelet counts as outlined below (see step 5). Certified blood-drawing personnel must notify the processing laboratory and the responsible research nurse when platelet samples have been drawn and notify the research nurse if unable to collect platelets as scheduled due to difficult sampling.

It is the responsibility of the laboratory staff to prepare platelet collection tubes, complete the Clinical Laboratory Test Requisition Form, and send specimens to the Brigham and Women's Hospital Clinical Laboratory.

It is the responsibility of the research nurse assigned to the research subject to determine on a daily basis the surveillance platelet protocol (see Cases A, B, C, and D in the "Procedure" section for specific guidelines) and to inform the certified blood-drawing personnel when to collect a blood sample for a platelet count.

It is the responsibility of the research nurse to obtain, document, and follow up results of the platelet count (if an "alarm value" as defined in "Procedure" section 6 below is met). It is the responsibility of the research nurse to instruct certified blood-drawing personnel to proceed with confirmatory testing, as outlined below. The confirmatory sample is obtained by the certified blood-drawing personnel in a manner identical to the initial sample; it will again be the responsibility of the research nurse to follow up results (within 1 hour of processing).

Procedure

1. Research nurse reviews protocol orders. Four cases have to be considered; the research nurse will determine which case applies to each different study protocol and with first intravenous (IV) insertion documents start time.

Case = Uninterrupted Infusion of Heparin

- Day 0 = Day of FIRST initiation of heparin infusion.
- Day 2 (4, 6, etc.) = 2 (4, 6, etc.) 24 hour-days elapsed since FIRST heparin initiation in the study.
- Blood sampling for platelet counts is to occur on days 2 (=2 × 24 hour-days of FIRST heparin initiation)—4, 6, 8, 10, 12, and 14 of heparin infusion. For subjects on non-24 hour-days, the sampling schedule should approximate this schedule. Longer studies do not require platelet sampling beyond day 14 of **continuous** heparin infusion.

Case B = Infusion of Heparin Has Been Started, Stopped 24 Hours or More, and then Reinitiated within the 0–14 Day Window

- Day 0 = Day of FIRST initiation of heparin infusion.
- Day 2 (4, 6, etc.) = 2 (4, 6, etc.) 24 hour-days elapsed since FIRST heparin initiation in the study.
- A platelet count is drawn anytime within the first 24 hours of heparin reinitiation and, following this, platelet counts continue to be drawn every 2 days until day 14 (day 0 = first time heparin is introduced) or until heparin is definitely stopped, whichever occurs first.

Case C = Infusion of Heparin Has Been Started, Stopped 24 Hours or More, with Reinitiation Happening after Day 14 and before Day 100

- Day 0 = Day of FIRST initiation of heparin infusion.
- Day 14 = 14 24 hour-days elapsed since FIRST heparin initiation in the study.
- Day 100 = 100 24 hour-days elapsed since FIRST heparin initiation.

- If heparin is discontinued and resumed within a window between days 14 and 100, platelets should be drawn anytime within the first 24 hours after heparin reintroduction. No further platelet counts are needed.

Case D = It Is Uncertain whether the Subject Has Received heparin in the past 100 Days

- In this case, the person should be considered as a case A and have platelets drawn on days 2, 4, 6, 8 10, 12, and 14 after heparin has been initiated (D0 will be considered then to be the first day they receive heparin in the present study).

2. Processing laboratory prepares EDTA tubes with printed patient identification label and delivers tubes to the applicable blood-drawing station.
3. If possible, the routine platelet surveillance sampling should occur in conjunction with routine blood draws, preferably DIRECTLY AFTER the routine blood sampling (i.e., prior to reinfusion). The timing of blood drawing should be scheduled near the beginning of wake-periods to allow, if necessary, sufficient time for follow-up blood tests.
4. Research nurse notifies monitoring staff of platelet samples scheduled each shift.
5. Certified blood-drawing personnel collect blood for platelet sample by performing the following steps:
    (a) Obtain identified, prelabeled 3-mL EDTA tube set up by processing laboratory (labeled with the printed patient identification label with subject's name, medical record number).
    (b) Confirm subject's name and date of birth off label with subject directly.
    (c) At the scheduled time of the platelet count blood draw, obtain the routine blood sample following blood sampling procedures.
    (d) Before reinfusion, draw an additional 1 mL using a clean and new 3-mL syringe.
    (e) Aliquot the 1-mL blood sample into the prepared tube.
    (f) Write date and time of collection on the patient identification label on the tube.
    (g) Immediately transport sample at **ROOM TEMPERATURE** to the processing laboratory.
    (h) Notify the processing laboratory that platelet sample has been drawn.
    (i) Notify research nurse that platelet sample has been drawn.
    (j) Document sample collection time and date.
6. Processing laboratory confirms information on the tube label, completes a Clinical Laboratory Test Requisition Form, packages with platelet sample in biohazard specimen bag, and sends to BW Clinical Laboratory.
7. In the event of difficulty in obtaining blood samples in the first quarter of the wake episode, certified blood-drawing personnel must notify research nurse and retry at next scheduled routine blood draw. After this first quarter of the wake period, to allow for follow-up time if necessary, the 1-mL sample for platelet surveillance is given priority over all other scheduled samples.
8. If the IV is being left with heparin still being infused in the subject without any blood draws occurring due to difficulty drawing, and if a platelet count was due in the past 24 hours and was not drawn due to the difficulty with the draws, then a venipuncture should be performed to obtain this platelet count.
9. The research nurse must document the results of the platelet count and compare the current platelet result and the value from the baseline sample (to be found in the orders with the heparin order).
    (a) If the platelet count is more than 50% of the baseline value, no further action is needed.
    (b) If the platelet count is *less* than 50% of the baseline value, referred further as "alarm value," follow the directions below.
10. If (b) occurs, the research nurse is responsible for instructing certified blood-drawing personnel to immediately collect a confirmatory 1 mL blood sample through the IV manifold in the same fashion as described in step 5, except without the preceding routine blood draw. Processing laboratory completes the requisition and sends the sample to BWH Clinical Laboratory as described in step 6 except that a "STAT" order annotation is made. The research nurse is then responsible for paging the project leader to inform him/her that a second platelet count has to be taken and for following up the result in a timely fashion.
11. In the event that the "alarm value" (less than 50% of the baseline value) is confirmed on the second platelet count (taken through the IV manifold as described in step 5), the research nurse is responsible for notifying the research project leader and the on-call study physician. The research nurse is also responsible for immediately discontinuing the heparin infusion (substituting 1/2NS at an identical infusion rate) and for evaluating the subject for any signs or symptoms of thrombosis or thromboembolism. Either the research nurse or a certified phlebotomist must draw a further 1 mL sample directly from the antecubital vein *contralateral* to the site of the IV. In an identical fashion to that described earlier, this sample is sent to the BWH Clinical Laboratory control for "STAT" platelet count. It is the responsibility of the research nurse to follow up the result of the platelet count in a timely fashion (within 1 hour). The research nurse must then clearly document the platelet result.
12. In the event that the third platelet count confirms the "alarm value," the research nurse must notify the research project leader and the on-call study physician who must evaluate the subject. As described in step 11, **such subjects are not to receive further heparin.**
13. The research nurse or a certified phlebotomist is responsible for obtaining a final blood sample for a heparin-induced thrombocytopenia PF-4 heparin antibody assay using the antecubital venipuncture technique on the arm contralateral to the IV manifold. The research nurse communicates as

soon as possible the order for platelet factor-4 heparin antibody test blood sample to processing laboratory. The processing laboratory will prepare a 3-mL citrate tube and BWH Clinical Laboratory Test Requisition Form with patient identification labels. The study physician follows up on the result and documents in the progress notes.

14. Decisions regarding the need for further medical evaluation and/or study termination are to be made by the study physician in discussion with the research project leader. A clear plan for medical follow-up of the subject must be in place before discharge is considered.
